# Inhibition of NF-κB Signaling Reduces the Stemness Characteristics of Lung Cancer Stem Cells

**DOI:** 10.3389/fonc.2018.00166

**Published:** 2018-05-17

**Authors:** Norashikin Zakaria, Narazah Mohd Yusoff, Zubaidah Zakaria, Darius Widera, Badrul Hisham Yahaya

**Affiliations:** ^1^Regenerative Medicine Cluster, Advanced Medical and Dental Institute, Universiti Sains Malaysia, Bertam, Penang, Malaysia; ^2^Cancer Research Centre, Institute for Medical Research (IMR), Kuala Lumpur, Malaysia; ^3^Stem Cell Biology and Regenerative Medicine, School of Pharmacy, University of Reading, Reading, United Kingdom

**Keywords:** NF-κB signaling, lung cancer, cancer stem cells, cancer migration, self-renewal

## Abstract

Cancer stem cells (CSCs) are a subpopulation of cancer cells that play a pivotal role in tumor development, invasion, metastasis, and recurrence. We and others have reported significant involvement of the NF-κB pathway in regulating CSCs of non-small cell lung cancer (NSCLC). In this study, we evaluated the effects of NF-κB inhibition on self-renewal, stemness, migration, and expression of genes involved in the epithelial to mesenchymal transition (EMT) and apoptosis resistance in lung CSCs. Different concentrations of the NF-κB inhibitor BMS-345541 (0.4, 4.0, and 10.0 µM), an inhibitor the NF-κB upstream kinase IKKβ, were used to treat both lung CSCs (CD166^+^CD44^+^, CD166^+^EpCAM^+^) and non-CSC NSCLC cells (CD166^−^CD44^−^, CD166^−^EpCAM^−^) in A549 and H2170 cell lines. We assessed the impact of BMS-345541 on the ability to form tumorspheres (self-renewal assay), expression of stemness genes (*SOX2, OCT4, NANOG, SCA-1*, and *KLF4*), migration, and expression of EMT and apoptosis-related genes. Inhibition of NF-κB by BMS-345541 effectively reduced the stemness, self-renewal, and migration capacity of lung CSCs. Moreover, expression of genes involved in the EMT (*SNAI1* and *TWIST*) and apoptosis resistance (*BCL-2, BAX*, and *BIRC5*) was significantly reduced following the treatments, suggesting that NF-κB inhibition is sufficient to prevent the EMT and induce apoptosis in lung CSCs. Our findings suggest that NF-κB inhibition could reduce the capability of CSCs to maintain their population within the tumor mass, potentially decelerating cancer progression, relapse, and chemotherapy resistance.

## Introduction

Emerging evidence suggests the presence of a subpopulation within tumors with the abilities to self-renew and to produce differentiated progeny. This subpopulation of cells, known as cancer-initiating cells or cancer stem cells (CSCs) (1), was first characterized in 1994 in leukemia (2). Their presence was later confirmed in many human carcinomas, including breast, brain, colon, and lung cancers (3–7). CSCs play a pivotal role in tumor development, invasion, metastasis, drug resistance, and recurrence (8). Previously, we isolated and characterized putative CSCs from non-small cell lung cancer (NSCLC) cell lines based on expression of specific cell surface markers (i.e., CD166, CD44, and EpCAM) (9). CSCs that were isolated based on the expression of combined CD166^+^/CD44^+^ and CD166^+^/EpCAM^+^ exhibited stemness *in vitro* and were able to form secondary tumors *in vivo*. The transcriptomic analysis of these populations of cells revealed a significant involvement of nuclear factor kappa-light-chain-enhancer of activated B cells (NF-κB) in regulating the putative lung CSCs.

The NF-κB family consists of five proteins: p65 (RelA), RelB, c-Rel, NF-κB1 (p105/p50), and NF-κB2 (p100/p52) (10, 11). These NF-κB proteins share a conserved N-terminal Rel homology domain, which enables nuclear translocation, DNA binding, and formation of homo- and heterodimers (10, 11). NF-κB proteins are expressed in the cytoplasm of most cell types, including differentiated cells, stem cells, and cancer cells (12, 13). In the inactive form, NF-κB homo- and heterodimers are bound to inhibitory IκB proteins (14) that mask the nuclear localization sequences (14). Activation of canonical pathways can be induced by many stimuli, including lipopolysaccharide and pro-inflammatory growth factors and cytokines [e.g., tumor necrosis factor alpha (TNF-α) or interleukin-1] (15). Upon activation, the IkB kinase (IKK) complex (IKKα, IKKβ, and IKKγ) phosphorylates the inhibitor IκB proteins at serine residues (e.g., 32 and 36 in IκBβ) and leads to their proteasomal degradation. Subsequent nuclear translocation of NF-κB and binding to its consensus sequences activate transcription of the respective target genes that regulate cellular proliferation, migration, and preventing apoptosis process.

Although aberrant NF-κB signaling has been associated with development and progression of various cancers, there is no detailed knowledge about the effect of this pathway on CSCs in lung cancer. In this study, the impact of NF-κB inhibition on the expression of genes associated with stemness, migration, and apoptosis of putative lung CSCs was evaluated. In addition, the effects of the NF-κB inhibitor BMS-345541 on the formation of tumorspheres (self-renewal) were also assessed. Finally, the influence of the blockade of NF-κB-mediated signaling on the migratory behavior of lung CSCs was evaluated using scratch-wound healing assay.

## Materials and Methods

### Materials and Cell Lines

BMS-345541 purchased from Sigma-Aldrich (St. Louis, MO, USA). Cell lines A547 (human lung carcinoma) and H2170 (human lung squamous cell carcinoma) purchased from the American Type Culture Collection (ATCC, Manassas, VA, USA).

### Cell Culture

All cells were cultured in RPMI-1640 medium (Gibco, Life Technologies, Foster City, CA, USA) supplemented with 10% fetal bovine serum (FBS) (Gibco) and 1% penicillin/streptomycin (Gibco) in a humidified incubator at 37°C and 5% CO_2_.

### Isolation and Characterization of Putative Lung CSCs

The putative lung CSCs and non-CSCs used in this study were isolated based on expression of specific CSCs markers (CD166, CD44, and EpCAM) (9, 16). Cells were detached with trypsin (Life Technologies, Carlsbad, CA, USA) and washed with phosphate buffer solution (PBS) containing 2% FBS (PBS with 2% FBS). A total of 1 × 10^7^ cells were incubated for 30 min (on ice and in the dark) with respective antibodies (Table 1). The unbound antibodies were washed away with PBS with 2% FBS through centrifugation. Prior to sorting, the labeled cell suspensions were filtered through a 40-µm cell strainer to obtain a single cell suspension. The labeled cells were sorted into CD166^+^/CD44^+^, CD166^−^/CD44^−^, CD166^+^/EpCAM^+^, and CD166^−^/EpCAM^−^ subsets using a fluorescence activated cell sorter (FACSAriaII, Becton Dickinson, Franklin Lakes, NJ, USA).

**Table 1:** List of antibodies used in cancer stem cells isolation.

Antibody	Clone	Isotype	Manufacture
CD166	3A4	Mouse IgG1, κ	BD Biosciences, San Jose, CA, USA
CD44	L178	Mouse IgG1, κ	BD Biosciences, San Jose, CA, USA
EpCAM	158206	Mouse IgG1, κ	R&D System, Minneapolis, MN, USA

### Quantitative Real-Time Polymerase Chain Reaction (qRT-PCR)

Total RNA was extracted from approximately 1 × 10^6^ cells using a Qiagen AllPrep RNA isolation kit (Qiagen, Limburg, Netherlands) according to the manufacturer’s instructions. The concentration and purity of the extracted RNA were determined using Nanodrop (Agilent Technologies, Santa Clara, CA, USA). The RNA samples were stored at −80°C for later use. Complementary DNA (cDNA) was synthesized from 3 µg of total RNA using the Transcriptor High Fidelity cDNA Synthesis Kit (Roche Applied Science, Mannheim, Germany) using a random hexamer primer and an anchored-oligo (dT) primer. Expression of selected genes was analyzed using an ABI StepOnePlus™ Real-Time PCR machine (Applied Biosystems, Foster City, CA, USA). The qPCR reaction was prepared using a Taqman^®^ gene expression assay (Applied Biosystems) and the primers listed in Table 2.

**Table 2:** List of Taqman^®^ gene expression primers.

Accession number	Gene symbol	Amplicon length (base pairs)
Hs01023894_m1	E-cadherin	61
Hs00983056_m1	N-cadherin	66
Hs00958111_m1	Vimentin	65
Hs00195591_m1	*SNAI1*	66
Hs02379973_s1	*TWIST1*	154
Hs01053049_s1	*SOX2*	91
Hs00999632_g1	*OCT4*	77
Hs04399610_g1	*NANOG*	101
Hs00358836_m1	*KLF4*	110
Hs00165656_m1	*SCA-1*	97
Hs00900055_m1	*VEGFA*	59
Hs00765553_m1	*CCDN1*	57
Hs00608023_m1	*BCL-2*	81
Hs00180269_m1	*BAX*	62
Hs04194392_s1	*BIRC5*	102
Hs02758991_g1	*GAPDH*	93

### Sphere Assay

The effect of NF-κB inhibition on CSC self-renewal was evaluated using a sphere assay. All cells (parental, CSCs, and non-CSCs of A549 and H2170 cell lines) were treated with different concentrations (0, 0.4, 4.0, and 10.0 µM) of BMS-345541 for 24 and 48 h. After each respective time point, the cells were trypsinized and 1.0 × 10^3^ cells/ml were cultured in 24-well ultra-low attachment plates (Corning, Corning, NY, USA) containing serum-free medium DMEM/F12 (Gibco) supplemented with 10 ng/ml fibroblast growth factor, 1% of B27 (optimized serum-free supplement), 20 ng/ml of epidermal growth factor, and 1% penicillin–streptomycin (all purchased from Invitrogen). Sphere size was assessed after 14 days. The sphere size was assessed by measuring the diameter (µM) of each formed spheres using ImageJ software.

### Migration Assay

Sorted and parental NSCLC cells were seeded at a density of 3–4 × 10^5^ cells/well in complete medium and grown overnight to a confluence of 90%. Subsequently, the cells were treated with colcemide (10 µg/ml) for 2 h. After incubation, a scratch was inflicted using a sterile 200 µl pipette tip, and gentle washing was carried out twice using PBS to remove debris. The cells were incubated with 1 ml of medium containing different concentrations of BMS-345541 for 24 and 48 h. Images of wound coverage were captured at ×10 magnification (Olympus IX 71; Olympus, Tokyo, Japan) at 0, 24, and 48 h. The coverage area was measured using ImageJ and the percentage of the migrated distance was calculated as:

$$\begin{array}{l} \text{Percentage of coverage area}=\\ \;(\text{area after 24}/\text{48 h}÷\text{initial area at }0\text{ h})\times \text{1}00. \end{array}$$

### Statistical Analysis

All data were expressed as the mean ± SD of three independent experiments. Comparison between treatment groups with the untreated group was performed using two-way analysis of variance (SPSS statistical package). *p*-Values of <0.05 were considered to be statistically significant.

## Results

### Inhibition of NF-κB Reduces the Expression Levels of Stemness Genes

**Figure 1:**
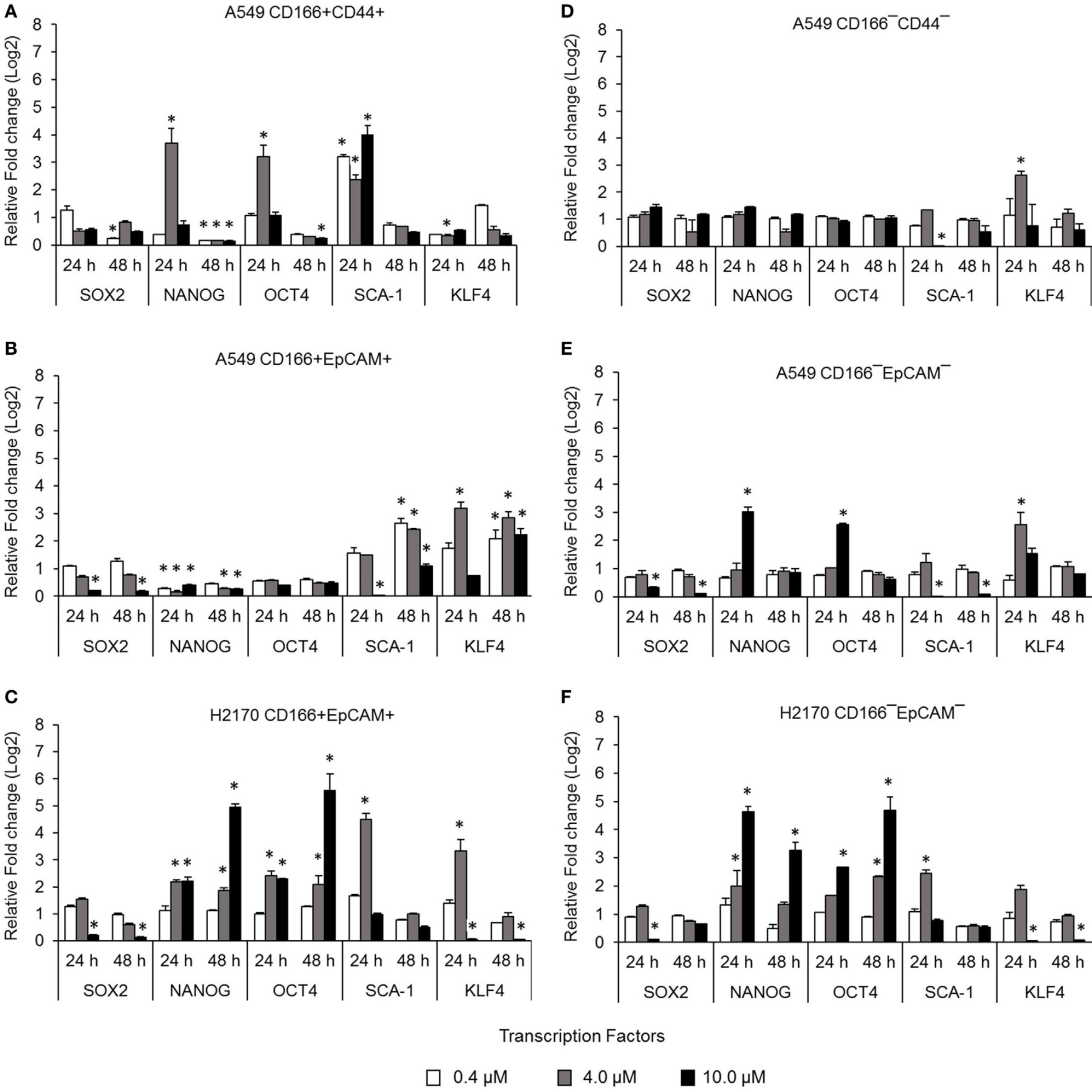
Effect of BMS-345541 on expression of stem cell transcription factors in lung cancer stem cells (CSCs). The graphs show the relative expression of stemness genes *SOX2, NANOG, OCT4, SCA-1*, and *KLF4* in **(A–C)** lung CSC and **(D–F)** non-CSC populations. The fold change was calculated using the 2^−ΔΔct^ formula, and *GAPDH* was used as the internal control. Graphs show fold change relative to the untreated sample. The results represent the mean ± SD of three replicates. The *p*-value was calculated using analysis of variance by comparing the treatment groups with the untreated group (*p < 0.05).

Cancer stem cells from NSCLC lines (A549 and H2170) were isolated based on expression of surface markers CD166, CD44, and EpCAM. The isolated CSCs (A549 CD166^+^CD44^+^, A549 CD166^+^EpCAM^+^, and H2170 CD166^+^EpCAM^+^) and their non-CSC counterparts (A549 CD166^−^CD44^−^, A549 CD166^−^EpCAM^−^, and H2170 CD166^−^EpCAM^−^) were treated with 0.4, 4.0, and 10.0 µM BMS-345541. The effect of the treatment was first evaluated by measuring the expression of stem cell transcription factors *SOX2, NANOG, OCT4, SCA-1*, and *KLF4* for both cell cancer and non-CSC populations (Figures 1A–F). Treatment with all concentrations of BMS-345541 for 48 h reduced the expression of *SOX2, NANOG, OCT4*, and *SCA-1* in A549 CD166^+^CD44^+^ cells. However, expression levels of the genes remained unaltered in the A549 CD166^−^CD44^−^ subpopulation except for *SCA-1* and *KLF-4*. In A549 CD166^+^EpCAM^+^ cells, the expression levels of *SOX2, NANOG*, and *OCT4* were reduced following treatment with 0.4, 4.0, and 10.0 µM BMS-345541. In the non-CSC subpopulation A549 CD166^−^EpCAM^−^, 10.0 µM BMS-345541 was needed to reduce the expression of *SOX2* and *SCA-1*. In H2170 CD166^+^EpCAM^+^ cells and the non-CSCs subpopulation (H2170 CD166^−^EpCAM^−^), treatment with 10.0 µM reduced the expression of *SOX2* and *KLF4* in the cells.

### NF-κB Inhibits the Expression of Genes Involved in the Epithelial to Mesenchymal Transition (EMT)

**Figure 2:**
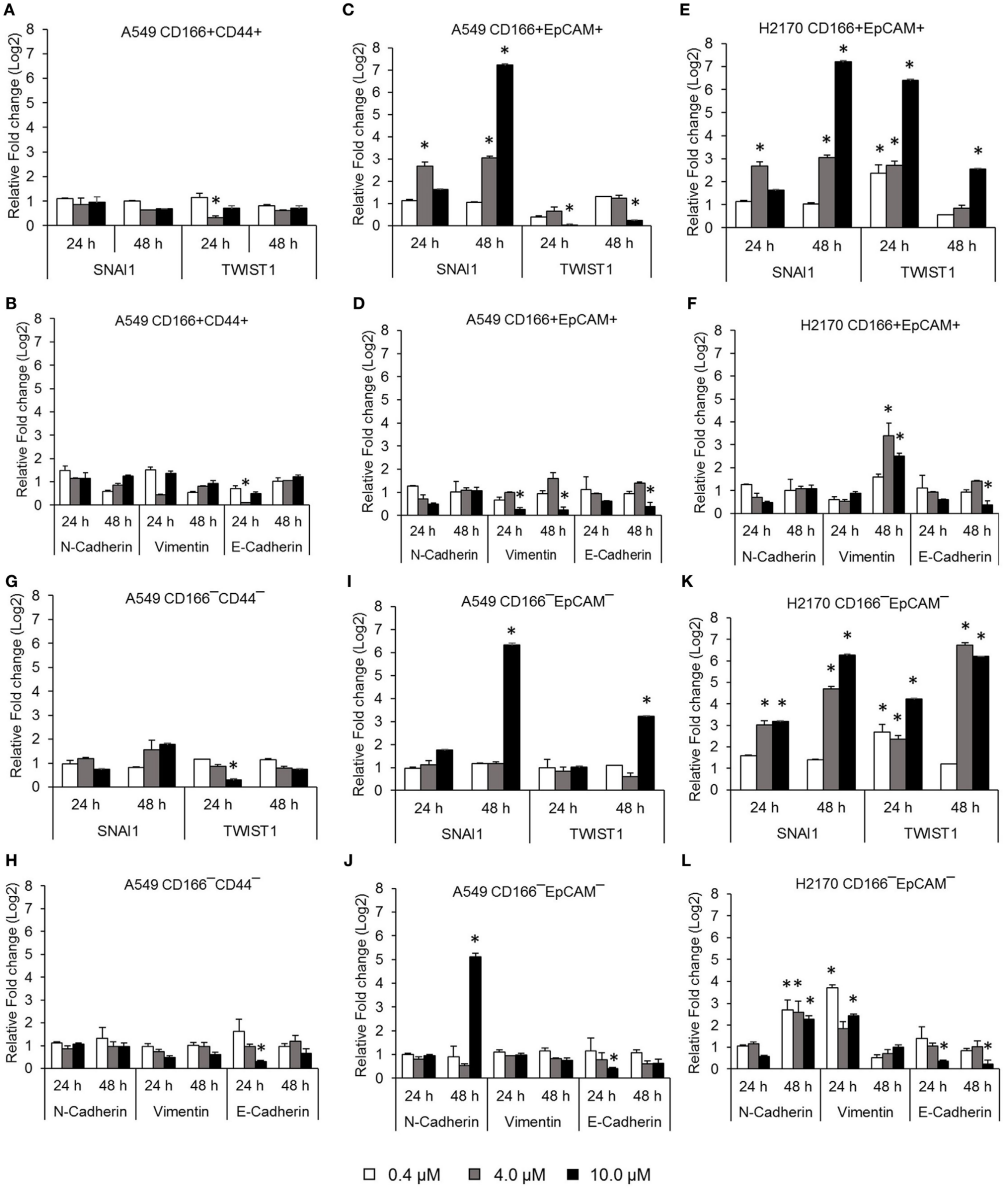
Quantitative real-time polymerase chain reaction results showing expression of genes involved in the epithelial to mesenchymal transition (EMT) process in lung cancer stem cells treated with different concentrations of BMS-345441. Relative expression of EMT switch genes (*SNAI1* and *TWIST1*), mesenchymal markers (N-cadherin and Vimentin), and the epithelial marker (E-cadherin) in **(A,B)** A549 CD166^+^CD44^+^; **(C,D)** A549 CD166^+^EpCAM^+^; **(E,F)** H2170 CD166^+^EpCAM^+^; **(G,H)** A549 CD166^−^CD44^−^; **(I,J)** A549 CD166^−^EpCAM^−^; and **(K,L)** H2170 CD166^−^EpCAM^−^ cells. The fold change was calculated using the 2^−ΔΔct^ formula, and *GAPDH* was used as the internal control. Graphs show fold change relative to the untreated sample. The results represent the mean ± SD of three replicates. The *p*-value was calculated using analysis of variance by comparing the treatment groups with the untreated group (*p < 0.05).

Activation of NF-κB is involved in several important processes in cancer, including regulating the expression of EMT transcription factors (17), inducing apoptosis resistance (18), and regulating the angiogenesis and proliferation of cancer cells. The effectiveness of the NF-κB inhibitor BMS-345441 in targeting lung CSCs was evaluated by its ability to reduce the expression of genes involved in those processes. Treatment with 0.4, 4.0, and 10.0 µM BMS-345441 did not exert any effect on expression of *SNAI1* and *TWIST1* (Figure 2A), and expression levels of mesenchymal markers N-cadherin and Vimentin and the epithelial marker E-cadherin were unchanged in A549 CD166^+^CD44^+^ cells (Figure 2B). Treatment with 10.0 µM BMS-345441 downregulated the expression of *TWIST1*, Vimentin, and E-cadherin in A549 CD166^+^EpCAM^+^ cells when treated for 24 and 48 h (Figures 2C,D). However, treatment of the cells with the same concentration of BMS-345441 increased expression of *SNAI1* when the treatment was prolonged to 48 h, but expression of N-cadherin was unchanged. In H2170 CD166^+^EpCAM^+^ cells, prolonged treatment with BMS-345541 for up to 48 h increased the expression of *SNAI1* (treatment with 10.0 µM) but increased the expression of *TWIST1* (treatment with 0.4, 4.0, and 10.0 µM) (Figure 2E). Expression of N-cadherin remained unchanged but expression of Vimentin increased following prolonged treatment, indicating that its expression is regulated by *SNAI1* (Figure 2F).

BMS-345441 treatment also affected the expression of EMT genes in non-CSCs. In A549 CD166^−^CD44^−^ cells, treatment with 4.0 and 10.0 µM BMS-345441 at 24 and 48 h slightly reduced the expression of *TWIST1*, but prolonged treatments increased the expression of *SNAI1* (Figure 2G). However, treatment did not have a significant effect on the expression of N-cadherin, Vimentin, and E-cadherin at both time point except for the expression of E-cadherin when treated with 10 µM BMS-345441 for 24 h (Figure 2H). In A549 CD166^−^EpCAM^−^ cells, treatment with 10.0 µM BMS-345441 for 48 h increased the expression of *SNAI1* and *TWIST1* (Figure 2I) and N-cadherin, but expression of Vimentin and E-cadherin were only slightly changed (Figure 2J). In H2170 CD166^−^EpCAM^−^ cells, treatment with 4.0 and 10.0 µM increased expression of *SNAI1, TWIST1*, N-cadherin, and Vimentin, but expression of E-cadherin was decreased (Figures 2K,L).

### NF-κB Inhibits the Expression of Genes Involved in the Apoptosis Resistance Process

**Figure 3:**
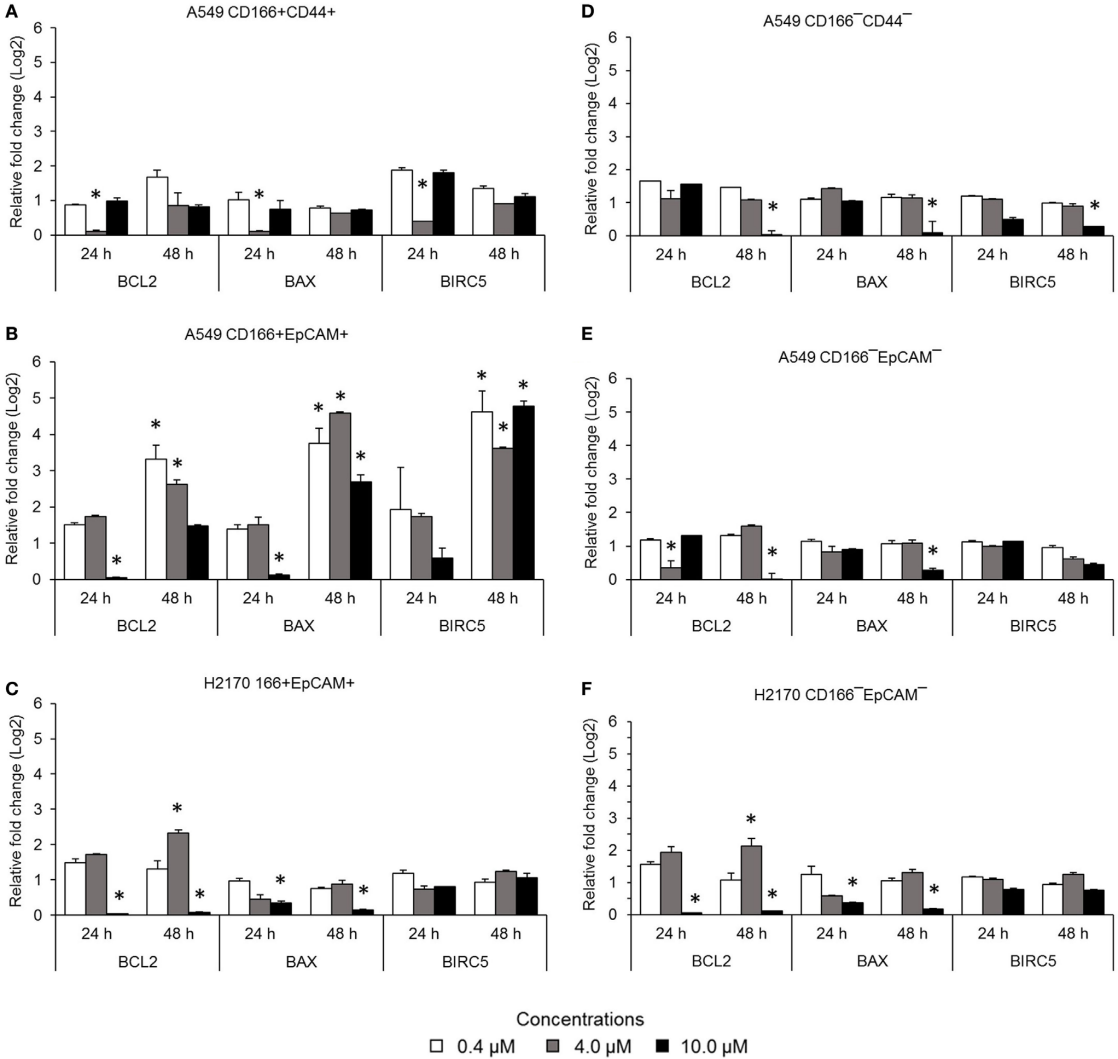
Expression of pro-apoptotic genes (*BCL-2* and *BIRC5*) and the anti-apoptotic gene *BAX* in lung cancer stem cells following treatment with different concentrations of BMS-345541. Relative expression of *BCL2, BAX*, and *BIRC5* in **(A)** A549 CD166^+^CD44^+^; **(B)** A549 CD166^+^EpCAM^+^; **(C)** H2170 CD166^+^EpCAM^+^; **(D)** A549 CD166^−^CD44^−^; **(E)** A549 CD166^−^EpCAM^−^; and **(F)** H2170 CD166^−^EpCAM^−^ cells. The fold change was calculated using the 2^−ΔΔct^ formula, and *GAPDH* was used as the internal control. The results represent the mean ± SD of three replicates. The *p*-value was calculated using analysis of variance by comparing the treatment groups with the untreated group (*p < 0.05).

The NF-κB pathway is involved in apoptosis resistance through activation of anti-apoptotic genes, including *BCL-2* and *BIRC5*. Expression levels of *BCL2, BAX*, and *BIRC5* in A549 CD166^+^CD44^+^ cells were downregulated when treated with 4.0 µM BMS-345541 for 24 h (Figure 3A). However, expression levels of *BCL2, BAX*, and *BIRC5* were unchanged when treated with 0.4 and 10.0 µM BMS-345441 compared to the untreated control. Expression of *BCL2* and *BAX* in A549 CD166^+^EpCAM^+^ cells treated with 10.0 µM BMS-345541 for 24 h was downregulated (Figure 3B). Prolonged treatment of the cells for up to 48 h led to increased expression of all three genes. Treatment with BMS-345441 reduced the expression of *BCL2* and *BAX* in H2170 CD166^+^EpCAM^+^ cells treated with 10.0 µM for 24 and 48 h (Figure 3C). In the three non-CSC subpopulations, treatment with 10.0 µM BMS-345441 was the most effective at inducing downregulation of *BCL2 a*nd *BAX* (Figures 3D–F). Expression of *BIRC5* in the cells remained unchanged following treatments with BMS-345441, except for A549 CD166^−^CD44^−^ cells, for which treatment with 10.0 µM BMS-345441 significantly downregulated expression of the gene (*p* = 0.035).

### NF-κB Inhibition Reduces Self-Renewal Capability of Parental and Lung CSCs

**Figure 4:**
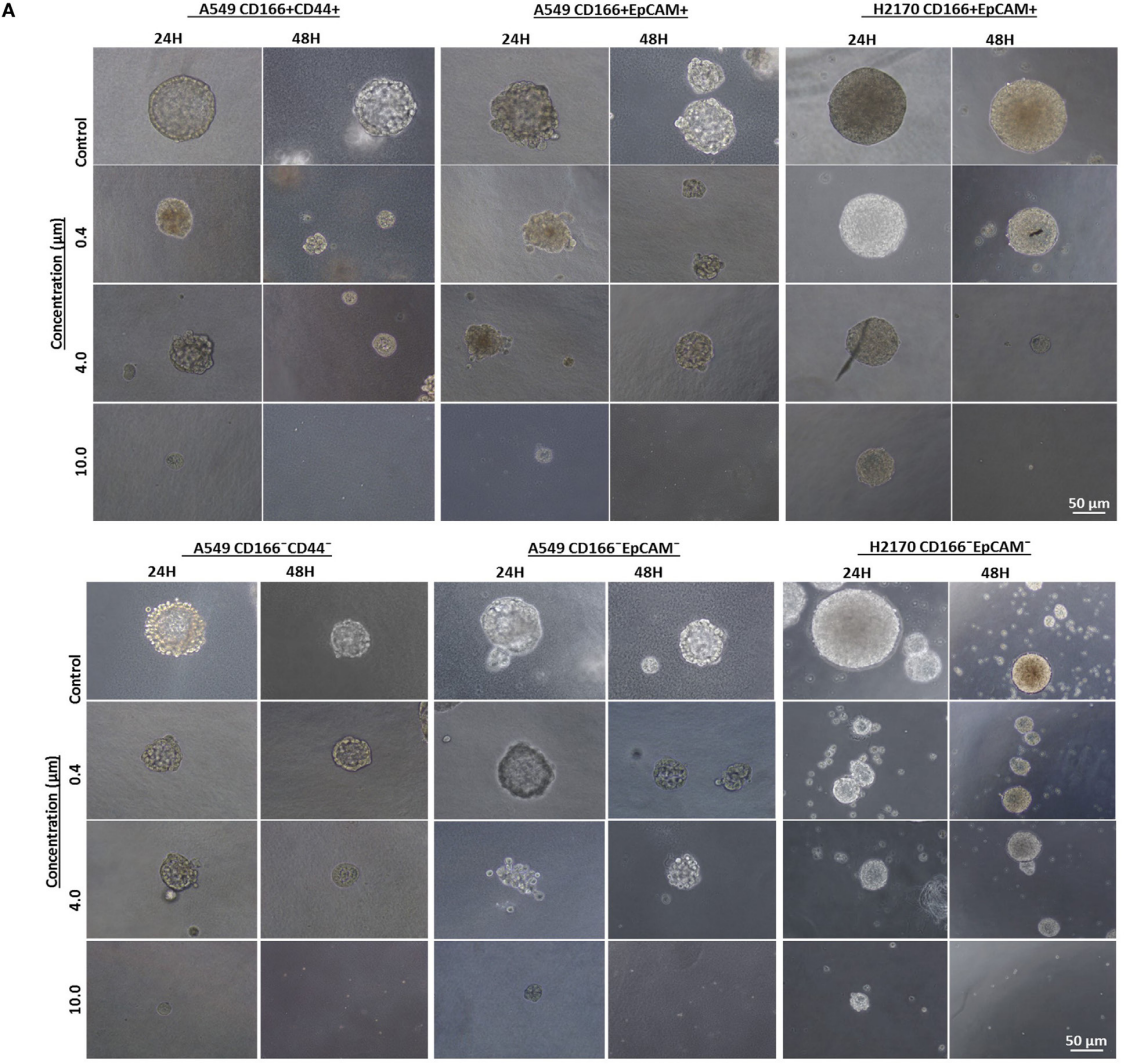

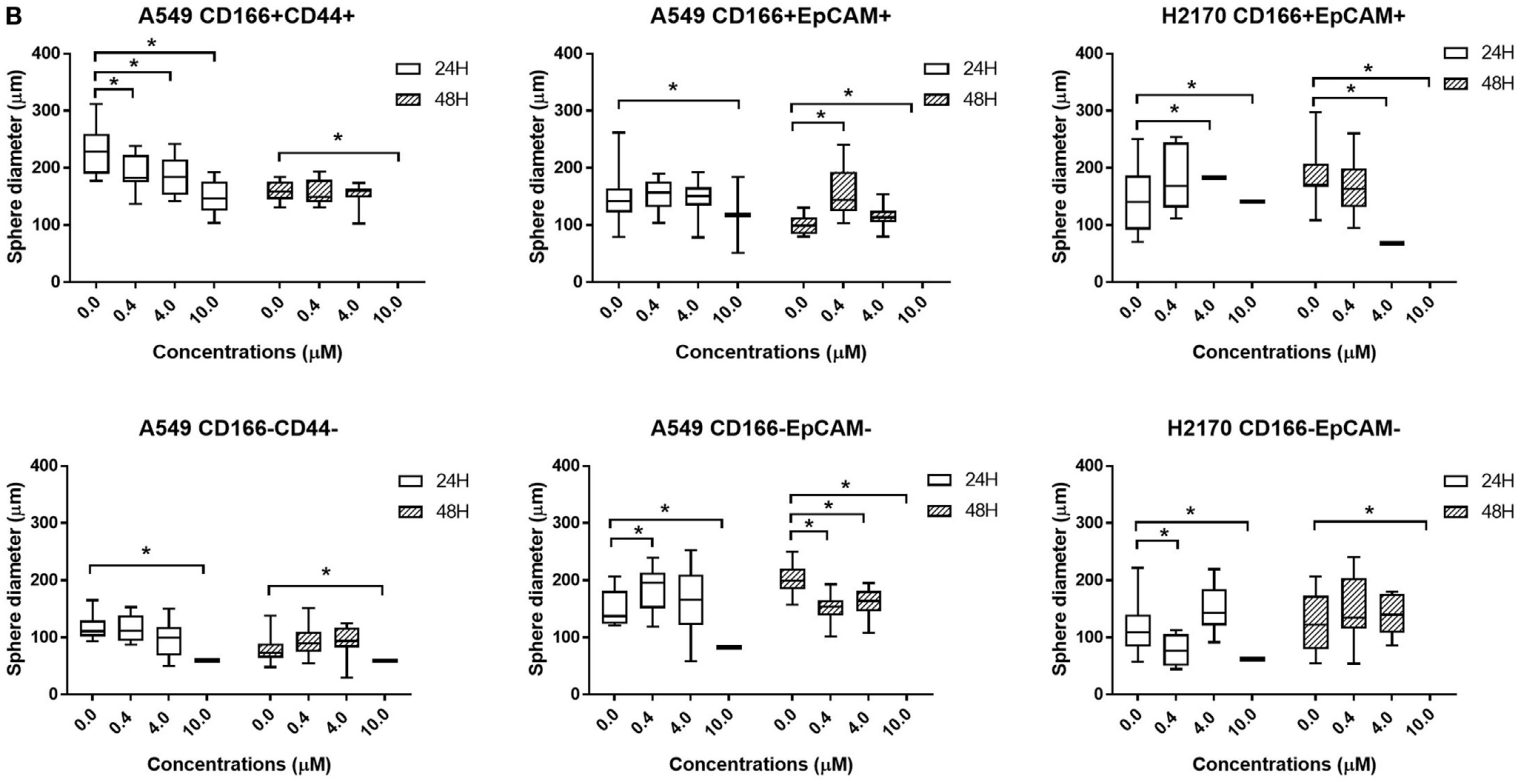
Effect of BMS-345541 on the sphere-forming capacity of lung cancer stem cells (CSCs). The cells were induced to form sphere following treatment with different concentrations of BMS-345541 for 24 and 48 h. **(A)** The images of the spheres formed following the treatments with BMS-345441 for lung CSCs and non-CSC counterparts. **(B)** Boxplots illustrate the sphere diameter (μm) for each cell type following the treatments. Data shown are representative of three independent experiments (*n* = 3). The *y*-axes show the sphere diameter (μm) and the *x*-axes show the concentration of BMS-345441 (0, 0.4, 4.0, and 10.0 µM) used in the treatment. Upper and lower boxplot margins represent the interquartile range, and the middle bar indicates the median. The whisker defines the range of values. The diameter of the spheres was measured using ImageJ analysis software. The bar above the boxes represents statistically significant differences *p < 0.05; two-way analysis of variance.

Self-renewal is an important characteristic of CSCs that helps maintain the CSC pool in the tumor. Therefore, the effectiveness of the NF-κB inhibitor BMS-345541 in targeting lung CSCs can be assessed by looking at its ability to reduce the sphere-forming capacity of lung CSCs. Following treatment with different concentrations of BMS-345441 for 24 or 48 h, the cells were induced to form spheres by culturing in sphere-forming medium. After 14 days, the diameter of formed spheres was measured. Regardless of the treatment status, both CSC and non-CSC populations had the ability to form spheres (Figure 4A). Treatment with BMS-345441 significantly reduced the sphere size of lung CSCs and non-CSCs in a concentration and time-dependent manner. Treatment with 0.4, 4.0, and 10.0 µM BMS-345441 for 24 h reduced the size of the spheres of A549 CD166^+^CD44^+^ cells (Figure 4B). For A549 CD166^+^EpCAM^+^ and H2170 CD166^+^EpCAM^+^ cells, the sphere size was reduced after treatment with 0.4, 4.0, and 10.0 µM BMS-345541 at 24 and 48 h (Figure 4B). The sphere size of non-CSCs was also reduced following treatment with different concentrations of BMS-345441 at different time points (Figure 4B).

### NF-κB Inhibits the Migration of Lung CSCs

**Figure 5:**
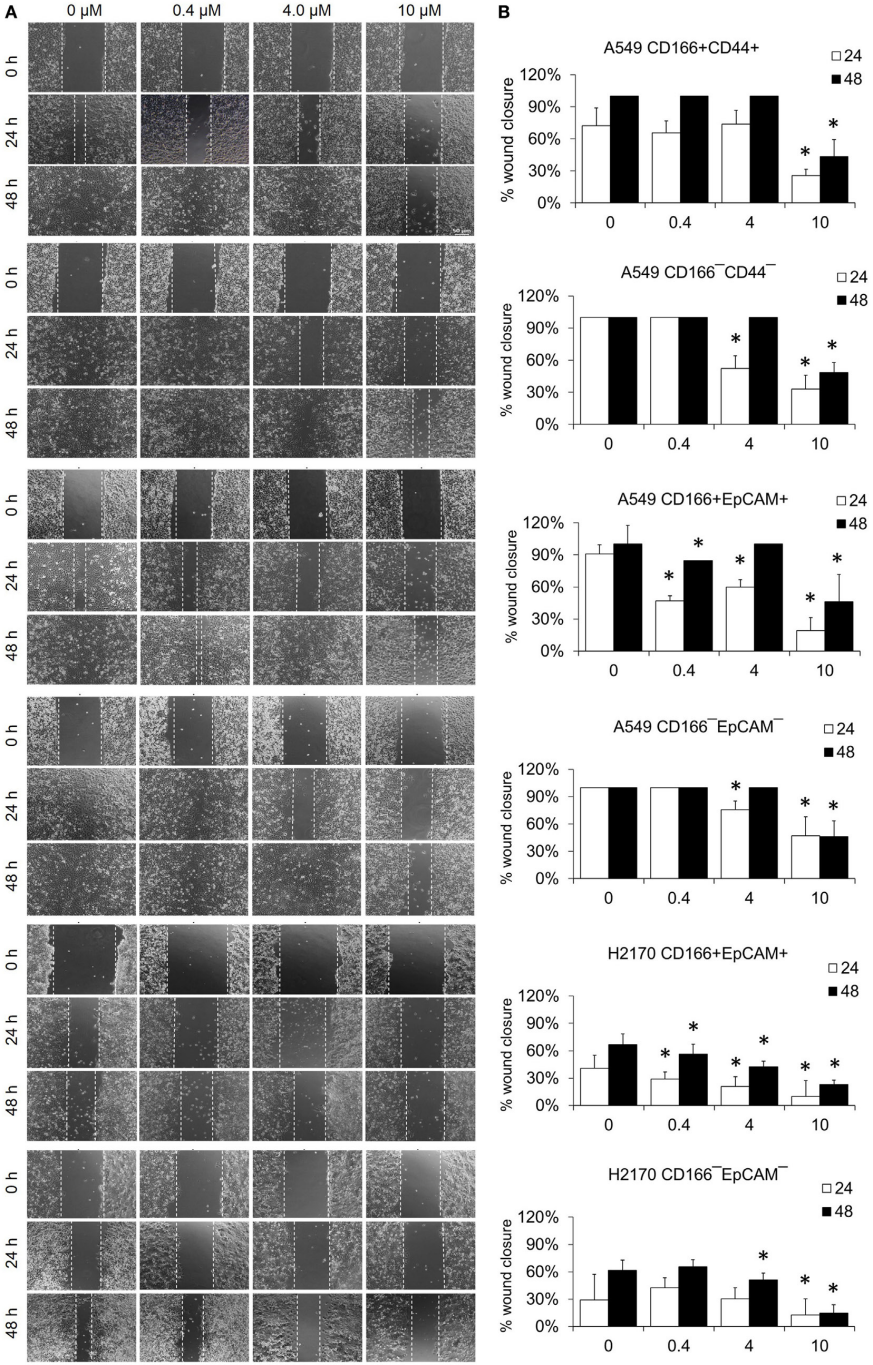
Effect of BMS-345541 on the migration of lung cancer stem cells of A549 and H2170 cells. Scratches were made and the cells were treated with different concentrations of BMS-345541. **(A)** Images of the wound area were taken at 0, 24, and 48 h after treatment. The wound areas were measured using ImageJ analysis software. **(B)** Percentage of migrated area [(area after 24/48 h ÷ initial area at 0 h) × 100%]. Data shown are representative of three independent experiments (*n* = 3). The *p*-value was calculated using analysis of variance by comparing the percentage of wound area for each treatment with that of the untreated group (*p < 0.05).

The migration assay was performed to determine the effect of the NF-κB inhibitor BMS-345541 in inhibiting the migration of lung CSCs and non-CSCs. Figure 5 shows images of the scratch-wound healing assay and the graph illustrating the percentage of wound closure. Treatment with 0.4, 4.0, and 10.0 µM BMS-345441 was effective in inhibiting the migration of A549 CD166^+^EpCAM^+^ (except 4.0 µM at 48 h) and H2170 CD166^+^EpCAM^+^ cells at 24 and 48 h. However, only treatment with 10.0 µM alone reduced the migration of A549 CD166^+^CD44^+^ cells when treated for 24 and 48 h. The migration of all three non-CSCs was inhibited by treatment with 4.0 and 10.0 µM, but the effect was time dependent. Taken together, these results show that higher concentration of BMS-345441 (10.0 µM) effectively inhibited the migration of CSCs and non-CSCs.

## Discussion

The presence of CSCs population in lung tumor is the key to treatment failure (19). This cell having the ability to self-renew and differentiate, which is the key to chemotherapy resistance thus makes it very difficult to eliminate the CSCs (20). NF-κB activation has been associated with the initiation and progression of several human cancers, including breast, cervical, prostate, pancreatic, and lung cancer (21–23). In lung cancer, a high level of NF-κB activity was detected in patients with SCLC and NSCLC, and its constitutive activity was associated with advanced stage and poor prognosis of patients (22, 24). Gene expression and bioinformatics result from our previous study demonstrated that NF-κB was highly associated with the CSCs gene signature (9) and inhibition NF-κB might resulted in reduction of the lung CSCs stemness and tumorigenic properties. The NF-κB molecule is regulated by the IKK complex (23, 25, 26), hence became the most suitable candidate for a therapeutic target for cancer treatments (27). BMS-345541, an IKK complex inhibitor, has been used to deactivate of NF-κB in various types of cancer, including melanoma and leukemia (28, 29). BMS-345541 at a concentration as low as 0.4 µM and up to 10.0 µM effectively inhibited activity of NF-κB (28–31). Therefore, in our study, BMS-345441 at three different concentrations (0.4, 4.0, and 10.0 µM) was used to treat lung CSCs, and the effects were evaluated against the expression of stemness, EMT and apoptosis genes, sphere-forming capability, and migration. The results indicate that BMS-345441 shows promising results in reducing sphere size, expression of stemness genes, and migration of lung CSCs as well as parental cancer cells. Moreover, expression of genes related to EMT and apoptosis was reduced following treatment, suggesting that inhibiting the NF-κB expression may help preventing EMT activities and induce apoptosis of lung CSCs.

Activation of NF-κB is one of the mechanisms that mediates self-renewal of CSCs (32, 33). Self-renewal is one of the main features of CSCs that helps maintain CSCs population in tumor. The effectiveness of NF-κB inhibition in targeting lung CSCs is evidence by the reduction of sphere-forming capacity of lung CSCs and non-CSCs. The ability to reduce the sphere-forming capacity reflects the self-renewal capability of those cells. Our finding is in parallel with reports for other types of cancer. For instance, inhibition of NF-κB using the plant-derived agent Triptolide reduced the colony- and sphere-forming capacity of pancreatic CSCs (34). Xiang et al. reported that NF-κB induced self-renewal of CD133^+^ ovarian CSCs and its inhibition has reduced the self-renewal capability of the cells (35).

Expression of several stemness genes, including *SOX2, OCT4, KLF4*, and *NANOG*, is an important indicator for the multipotent characteristic of CSCs (9, 36). Our study demonstrated that inhibition of NF-κB reduced the expression of stem cell transcription factors *SOX2, NANOG*, and *OCT4* in CSCs of A549 cells and expression of S*OX2, SCA-1*, and *KLF4* in CSCs of H2170 cells. The roles of transcription factors in maintaining the stemness and tumourigenicity state of the CSCs have been reported previously. For example, *SOX2, OCT4*, and *NANOG* were found to be overexpressed in several cancers, including breast, prostate, and oral squamous cell carcinoma, and their expression levels were associated with tumor transformation, tumourigenicity, and tumor metastasis (37–39). Unlike in embryonic stem cells where these transcription factors mostly control differentiation of the cells, overexpression of *SOX2, OCT4*, and *NANOG* in CSCs was reported to modulate signaling pathways involved in the inhibiting apoptosis (40, 41). Therefore, reduction in the expression of these transcription factors indicates that the cells have lost their multipotent characteristics, including the self-renewal capability, and the reduction also likely induces apoptosis of the cells. The effect of NF-κB in regulating the expression of stemness genes was not consistent in every cell, which indicates differently response to the treatments. This is likely due to the heterogeneous population of CSCs as a result from asymmetric division which generates diversity within the cancer (42). NF-κB activation has been associated with invasive and metastatic capabilities of cancer and CSCs. The cancer cells invasion and metastasis process occur when the epithelial cancer cells loss its adherent properties and acquire the mesenchymal phenotype which then allow the cells to migrate (43, 44). This process is known as EMT. EMT process is activated by transcription of its genes including *SNAI1* (45, 46), *TWIST1* (47, 48), Slug, and *ZEB1/2* (49) [reviewed in Ref. (17)] and its activation is demonstrated by the loss of epithelial marker (E-cadherin) expression and gain of mesenchymal marker (Vimentin and N-cadherin) expression (49, 50). The roles of the EMT in CSCs and cancer cell migration and metastasis suggesting that inhibiting the EMT process could result in reducing migration and metastatic potential of CSCs. Our results demonstrated that the migration capability of CSCs was reduced following the treatment with IKK kinase inhibitor, BMS-345541. This finding was in agreement with previous finding in NSCLC where inhibition of NF-κB has resulted in suppression of EMT transcription factors TWIST1, SNAI1, Slug, and ZEB2 and prevented the invasion and metastasis of the cells (51). Although our migration data show that NF-κB inhibition have significantly reduced CSCs migration, the expression of EMT genes was not in agreement with the data.

The gene expression data demonstrated that BMS-345541 treatment reduced the expression of *TWIST1* in lung CSCs populations A549 CD166^+^CD44^+^, A549 CD166^+^EpCAM^+^, and H2170 CD166^+^EpCAM^+^but increased the expression of *SNAI1* in A549 CD166^+^EpCAM^+^ and H2170 CD166^+^EpCAM^+^ cells. The same result was seen in the non-CSC populations A549 CD166^−^EpCAM^−^ and H2170 CD166^−^EpCAM^−^. Although both *SNAI1* and *TWIST1* control activation of the EMT process, these genes are activated through different pathways. SNAIL family genes are activated through the NF-κB pathway, but TWIST family genes are activated through the Ras/Raf/ERK pathway (52, 53). *SNAI1* upregulation *via* NF-κB occurs through phosphorylation of IKKα, which then activates NF-κB/p65 dimer and stimulates transcription of *SNAIL* (54). TheBMS-345541 molecule is specifically targets the IKKβ subunit of the IKK complex, the IKKα subunit remain free to undergo phosphorylation, activate the NF-κB/p65 dimer and increase *SNAIL* expression. However, the upregulation of *SNAIL* alone is not enough to activate the EMT because its activation is controlled by sequential activation of several activators, including *TWIST, ZEB*, and *Slug* (53).

The NF-κB activation also involved in apoptosis resistance in cancer cells (28). This event occurs through overexpression of anti-apoptotic genes (14). The anti-apoptotic proteins include BCL-2, BCL-X_L_, and MCL-1, and BIRC5 whereas the pro-apoptotic proteins include BAX and BAK as well as the BH3 domain molecules (e.g., Bid, Bim, Bik, Noxa, and Puma) (55). In our study, inhibition of NF-κB downregulated expression of the anti-apoptotic genes *BCL-2* and *BIRC5*. Deactivation of NF-κB was reported to be associated with downregulation of *BCL-2 via* a mechanism of caspase-3 activation that mediates *BCL-2* cleavage that later induces apoptosis activity (29). BMS-345541 in the low micromolar range (1–2 µM/l) has been reported to induce apoptosis in leukemia cells (56). A higher concentration (10.0 µM) of BMS-345541 was found to induce apoptosis of melanoma cells (28). A lower concentration (2–5 µM) also resulted in induction of a moderate pro-apoptotic effect (28). The ability of BMS-345541 to induce apoptosis was also seen in combination with TNF-α (28).

## Conclusion

Results of this study indicate that NF-κB is a promising molecular target for lung CSCs, as the deactivation of NF-κB using the kinase inhibitor BMS-345541 effectively reduced the stemness, self-renewal, and migratory capabilities of lung CSCs. These effects limited the capability of CSCs to maintain their population, and in theory this treatment might reduce cancer resistance to therapy and limit cancer progression. This finding may be a key to solving some major challenges in cancer therapy, namely cancer relapse and chemotherapy resistance, by eliminating CSCs. Future studies are needed to further elucidate the molecular machinery responsible for the effects of the inhibitor and to determine how it effects on other pathways that regulating cancer and normal stem cells. The cytotoxicity of the kinase inhibitor also must be evaluated specifically in lung cancer patient samples because it may have an unknown adverse effect. BMS-345541 has the potential to be developed into a specific anti-CSC therapy, but its use alone did not show consistent effects in targeting all different phenotypes of lung CSCs.

## Author Contributions

BHY and NZ designed, conducted the experiment and analyzed the data: NZ, BHY, DW, NMY, and ZZ contributed in writing and editing of the manuscript.

## Conflict of Interest Statement

The authors declare that the research was conducted in the absence of any commercial or financial relationships that could be construed as a potential conflict of interest.
